# Inflammasomes and Natural Ingredients towards New Anti-Inflammatory Agents

**DOI:** 10.3390/molecules21111492

**Published:** 2016-11-08

**Authors:** Patrick Dutartre

**Affiliations:** Laboratory BioperoxIL, Faculty of Sciences SVTE, University of Bourgogne Franche Comté, 6 Bd Gabriel F-21000 Dijon, France; patrick.dutartre@u-bourgogne.fr; Tel.: +33-380-393-268

**Keywords:** inflammasomes, natural antioxidants, inflammation

## Abstract

Inflammasomes are a family of proteins in charge of the initiation of inflammatory process during innate immune response. They are now considered major actors in many chronic inflammatory diseases. However, no major drug focusing on this target is currently on the market. Among the various approaches aiming to control this major metabolic pathway, compounds aiming to modify the intracellular antioxidant profile appear to be promising. This can be obtained by “light” antioxidants able to induce natural antioxidant response of the cell itself. This review will give an overview of the current available information on this promising pharmacology approach.

## 1. Introduction

Innate immunity is under the control of Pattern Recognition Receptors (PRRs) able to recognize Pathogen-Associated Molecular Patterns (PAMPs) originating from external pathogens, and/or Danger-Associated Molecular Patterns (DAMPs) originating from endogenous stress. The initial answer is the induction of inflammation, which is a normal biological process leading to the elimination of the initial aggressor. Inflammation can be eventually followed by stimulation of adaptive immunity. The first step of inflammation is the production of the pro-inflammatory cytokines Interleukin-1β (IL-1β) and interleukin-18 (IL-18) and the immunoregulatory cytokines type 1 interferon-α and β (INFα and INFγ) when PAMPs are externally recognized. Altogether, these cytokines have demonstrated a major role in innate and adaptive immunity. The pro-inflammatory cytokines IL-1β and IL-18 are produced under the control of inflammasomes, a family of proteins described for the first time in 2002 [1]. Inflammasomes were initially reported for their role in rather marginal or quite benign pathologies such as periodic fevers and gout but are now considered key actors in many chronic inflammatory diseases. Their roles in metabolic, neurologic and autoimmune diseases as well as in the initiation, control and dissemination of cancer are the subject of recent elegant reviews [2,3,4,5,6,7,8,9,10,11]. Reactive oxygen species (ROS) are a major regulator of this metabolic pathway [12] so compounds able to modify intracellular redox equilibrium may have a strong pharmacological activity on innate and/or adaptive immunity.

With the exception of mifamurtide for the co-treatment of osteosarcoma [13] in humans, no marketed compound described to interact with the inflammasomes pathway is available on the market. Mifamurtide is available in Europe only. This drug is a lipopeptide analogue of muramyl dipeptide developed as an activator of macrophages by interaction with intracellular PRRs [14].

At the clinical level, inflammatory symptoms such as fevers, tremors and pain are efficaciously controlled by anti-inflammatory agents acting on the secondary steps of inflammatory response. However, these drugs may be expensive (i.e., recombinant drugs), and are always associated with high morbidity (i.e., steroids [15] or non-steroids [16]), especially after chronic administration. Furthermore, at the patient’s level, a strong need for natural and less expensive preventive or curative treatments is noticed so new therapeutic options are urgently required. This review will summarize the current scientific information on inflammasome pathway and describe the modulation of this target with natural anti-oxidants able to act directly or indirectly on the activation of the inflammasomes.

## 2. PRR Families

Although a precise landscape of PRRs families remains to be definitively established and stabilized, five families of PRRs are currently described (Table 1). The PRRs able to respond to PAMPs are Toll-like Receptors (TLRs) and C-type Lectin Receptors (CLRs). They are expressed at the cell membrane level and in the endosome membrane. They are able to stimulate the Nuclear Factor-kappa B (NF-κB) pathway with global cell activation, and, in particular, synthesis of components of the inflammasomes and production of the type 1 interferons. The PRRs able to respond to DAMPs and PAMPs are the inflammasome Nucleotide-binding domain and Leucin-rich repeat Receptors (NLRs), Retinoic acid-inducible gene Like Helicase Receptors (RLHs) and Absent In Melanoma-like receptors (AIMs). NLRP3, who belongs to the NLR family, is the most studied inflammasome. NLRs, RLHs and AIMs are expressed intracellularly. They are responsible for the production of pro-inflammatory cytokines IL-1β and IL-18 and are deeply involved in the induction of a particular form of cell death named pyroptosis. This non-apoptotic cell death depends on the activation of caspase-1 and/or 5 and/or 11 with major modifications of cell membrane integrity leading to rapid cell death and further production of pro-inflammatory cytokines. However, the precise biochemical mechanisms and final role of this particular cell death remain to be defined [17]. The inflammasomes are activated by many ligands including peptidoglycans ribonucleic acids, and ROS are reported to be a major regulator of this metabolic pathway [18].

**Figure 1:**
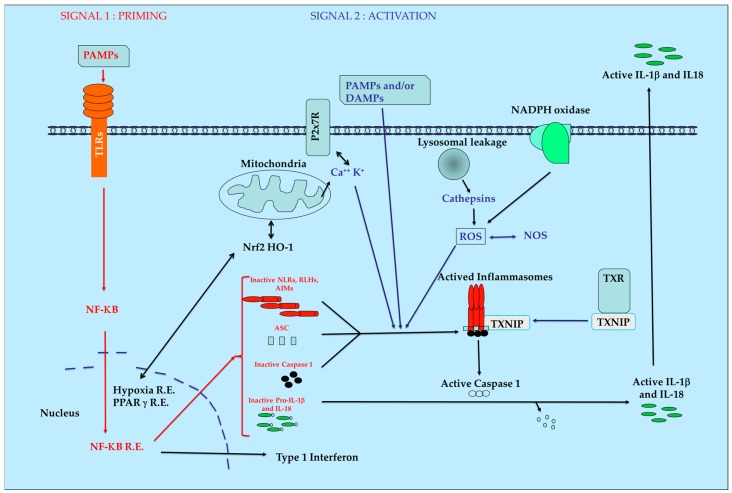
Simplified view of inflammasomes pathway. AIM: Absent In Melanoma receptors, ASC: apoptosis-associated speck-like protein, DAMPs: Danger-Associated Molecular Patterns, HO-1: Heme-Oxygenase 1, IL1-β: Interleukin 1 *béta* IL-18: Interleukin 18, NF-κB: Nuclear Factor-kappa B, NLRs: Nucleotide-binding domain and Leucin-rich repeat Receptors, NOS: Nitric Oxyde Species, Nrf2: Nuclear factor erythroid 2–Related Factor 2, PAMPs: Pathogen-Associated Molecular Patterns, PRRs: Pattern Recognition Receptors, R.E.: Responsive Elements, RLHs: Retinoic acid-inducible gene Like Helicase Receptors, ROS: Reactive Oxygen Species, TXNIP: Thioredoxin Interacting Protein, TXR; thioredoxin.

The inflammasome proteins are produced in cells in an inactivate form who must be activated by superstructure assembly and maturation processes leading to a rapid biological answer. This process can be also observed during coagulation, knowing that the set of genes for both pathways were reported to derive from the same ancestral cluster [19]. After stimulation by PAMPs and/or DAMPs and with complementary signals, the inactive proteins of the inflammasomes will be aggregated. This induces the activation of the ATPase function leading to the final activation of protease caspase-1. Then, the maturation of inactive pro-IL1 β and pro IL-18 into their active final form occurs. Pyroptosis can be also induced according to the nature and intensity of initial stimuli. A simplified model of inflammasome pathways is presented in Figure 1.

Production of ROS by mitochondria in combination with thioredoxin (TXN), Thioredoxin Interacting Protein (TXNIP) and heme-oxygenase 1 (HO-1) are key regulators of intracellular redox with major importance during initiation and maintenance of inflammatory pathologies and for mitochondria homeostasis [20]. The production of cathepsins after lesions of the phagosome and modification of the NADPH oxidase equilibrium are also directly or indirectly involved in the functions of the inflammasomes. The influence of another actor such as nuclear factor erythroid 2–related factor 2 (Nrf2) able to induce natural anti-oxidant defense is also evidenced [21]. If the initial equilibrium cannot be re-obtained rapidly after this initial burst, a situation of chronic inflammation occurs with development of fibrosis [7] necrosis [22] and pain [23].

## 3. Natural Anti-Oxidants and Inflammasomes

The appearance of a new biological target always leads to a wave of patents and publications in fundamental and applied domains. The inflammasomes do not escape to this tradition.

Considering patents, one major limitation for the use of the inflammasomes as an argument for originality is the patent from Tschopp and Martinon [24] in which the authors described the method for identification of inhibitors of the inflammasomes. This patent rendered the target itself extremely difficult to use without risking an anteriority problem. However, a few published patents identified by “inflammasomes” in the title or the summary can be identified knowing that the word “inflammasomes” itself is not in the thesaurus of the patent database. Table 2 summarizes the main results for compounds or compositions with antioxidant capacity claimed as useful for the treatment of inflammatory disorders involving direct or indirect effect on the inflammasomes.

In the scientific literature, numerous compounds with strong or weak antioxidant properties are described to be active on the inflammasome pathway.

Citral is an acyclic unsaturated monoterpene aldehyde naturally present in the Chinese herbal medicine *Litsea cubeba*. This compound has antioxidant properties [37] and was recently described as an anti-inflammatory agent with direct effects on NLRP3 activation signals but not on the priming step. The antioxidant signal Nrf2 is claimed to be strongly enhanced by the administration de citral in vivo to mice in a model of lupus nephritis [38].

Schisandrin B extracted from *Schisandra chinensis*, another Chinese medicinal herb, is one of the various dibenzocyclooctadiene antioxidant lignin derivatives present in this herb [39]. The publication of Leong and Ko [40] shows that in vitro as well as in vivo schisandrin A is able to stimulate Nrf-2 for the expression of thioredoxin with concomitant inhibition of inflammasome functions. A rather large series of published papers are available on these antioxidant drugs reported to be global stimulators of physical capacity and memory with cardiovascular protection and influence on apoptosis [41]. However, until now, no precise demonstration of the underlying mechanism of action is available.

In the vast family of polyphenols, numerous, if not all compounds, are described as antioxidants [42] with a large panel of pharmacology activities. The elegant review of Leyva-López et al. on the potentialities of the flavonoid family in various domains of inflammatory diseases and immunity disorders is of particular interest [43]. Globally, these compounds are able to decrease ROS and NOS production as well as the COX-1 and COX-2 activities. This may lead to control of acute inflammation and pyroptosis development.

Quercetin is a reference as an antioxidant flavonoid with a wide range of pharmacological effects. The effect of a series of antioxidants including quercetin on fructose-induced toxicity on hepatic function in rodents was recently published [44]. Inhibition of TXNIP with a final decrease of the functioning of the inflammasomes, an impairment of inflammation and an inhibition of lipid deposit confirm the great interest of this type of antioxidants on hepatic inflammatory diseases. Rutin, the 3-glucorhamnoside derivative of quercetin, was also studied alone [45] in comparison with quercetin [46] as modulators of activities of NLRP3. In vivo in rats receiving ethanol and cerulein, the parenteral administration of rutin significantly ameliorates pancreatic markers. More specifically, mRNA and protein expression of caspase-1, ASC and NLRP3 were decreased, showing the influence of rutin on the pathway of the inflammasomes without precise explanation of this beneficial effect.

Resveratrol is certainly the most well-known natural phenol and is reported to be active in prevention and or treatment of various pathologies of the cardiovascular and central nervous system, to delay aging process, and to interfere with cancer initiation and progression. Since 2013, a series of publications reporting interactions with the pathway of inflammasomes have appeared. In vitro *cis*-resveratrol was reported in normal human macrophages to decrease P2X7 receptor concomitantly with a decrease of ROS production [47]. In a more focused experiment, resveratrol was reported to interact with NLRP3 assembly in murine macrophages after mitochondrial damage. The selectivity of action of resveratrol on NLRP3 was demonstrated by the lack of effect on IL-1β production after stimulation with flagellin or dsDNA [48]. In vivo experiments in a rat heart ischemia/reperfusion model [49] showed that pre-treatment with resveratrol decreased, in a dose dependent way, the infarct volume and fibrosis of the myocardium. NLRP3, caspase-1, IL-1β and IL-18 expression were also decreased. In food-induced inflammation, two studies are available. The first in mice with hepatic inflammation after administration of a high fat regimen leading to induction of obesity showed a reduction of inflammatory markers with modification of the pathway of sirtuin and the activation of the inflammasomes [50]. The second in rats receiving a cholesterol-enriched diet in combination with vitamin D2 [51] in which circulating IL-1β and expression of inflammasomes components at the vascular level decreased after resveratrol administration. In this experiment, some other activities of resveratrol were also reported such as hypolipidemic and antioxidant effects (decrease in malonaldehyde (MDA), increase of superoxide dismutase (SOD)). Altogether, these results cannot really conclude on a univocal direct effect of resveratrol on the inflammasome pathway.

Apigenin, a flavonoid with a wide range of activities, is now considered to act at various steps of the pathway of the inflammasomes through the decrease of NF-κB activation, the inhibition of extracellular signal-regulated kinases 1/2 (ERK1/2), activation and disruption of NLRP3 assembly in macrophages [52]. In vivo, a decrease of activation of IL-β and NLRP3 activation was reported during a depressive behavior induced in the mice by stress, in parallel with an induction of expression of the peroxisome proliferator-activated receptors γ (PPARγ) expression [53]. These results are correlated with the observation of the relation between intracellular redox state and PPARγ [54]. Therefore, antioxidant agents able to act on a nuclear receptor function may have indirect effect on the pathway of the inflammasomes in relation or not with their initial antioxidant potential.

Curcumin extracted from *Curcuma longa* is used in traditional Chinese medicine and in Ayurveda medicine from India. The powerful antioxidant activity with the inhibition of cyclooxygenase 2 (COX-2), the lipoxygenase (LO), and the inducible Nitric Oxyde Synthase (iNOS) is described as the mechanism of action of this compound [55]. Among the vast bibliography available on curcumin, two publications are of particular interest. The dual effect of curcumin on mesothelium malignant cells of human or murine origin is somewhat surprising because the compound was able to induce in parallel the pyroptosis of cancer cells and protection against inflammation. Pyroptosis was observed in cancer cells with caspase 1 stimulation without processing of pro-IL-1β due to inhibition of the NF-κB pathway [56]. When curcumin is added to SH-SY-5Y cells, a human neuronal cell line, and treated with glutamate, a decrease of TXNIP was measured with the inhibition of NLRP3 expression and the IL-β activation [57]. In the same publication, an oral administration of curcumin gave protection against the infarct and the neuronal damage induced by the occlusion of cerebral arteries with an inhibition of NLRP3 expression in the hippocampus.

Epigallocatechin, a flavanol derivative, is also highly active on the pathway of the inflammasomes. In vivo in a model of nephropathy in the rats, the compound has acted indirectly on NLRP3 by HO-1 induction [58]. Expression of AIM-2 was also decreased in human epidermal cells by acting at two different levels, firstly by suppression of interferon priming, secondly by inhibition of oligomerization of ASC [59]. Three other publications using the same compound have confirmed its influence on the global cell metabolism leading to modification of the pathway of the inflammasomes through modulation of the antioxidant machinery [60,61,62].

Derivatives of phenol were also studied. Ferulic acid is a strong antioxidant [63] and a decreased expression of the inflammasomes with a concomitant increase of antioxidant status of the cells was reported in joint homogenates of rats receiving monosodium urate injections. Docking analysis claimed a “significant efficiency” against pro-caspase-1, NF-κB, ASC, and NLRP3 who seemed to demonstrate a physical interaction between ferulic acid and its putative target [64].

Coumarins are also especially well known for their capacities as anti-inflammatory and anti-oxidant activities. They are also especially appreciated by chemists and pharmacologists as a starting point aiming to obtain original pharmacological compounds [65]. In a model of cerebral ischemia induced in the rat by cerebral artery occlusion, an oral administration of umbelliferone has ameliorated clinical symptoms in parallel with the modification of PPARγ expression and a decrease of TXNIP in relation to NLRP3 activation [66].

The anthraquinones are a family of compounds present in a large number of oriental traditional medicines having antioxidant properties [67]. Emodin has been described to selectively decrease NLRP3 response to nigericin, ATP and urea crystals in vitro and decrease the severity of endotoxic shock in rodents [68].

The family of xanthone is especially numerous and more than 200 different chemical structures are reported in the literature with powerful activities including anti-inflammatory properties. Mangiferin, a glycosylated derivative of anthraquinone, is a powerful anti-inflammatory agent (see [69] for review) that behaves as a strong antioxidant compound as demonstrated by protection of the carbonylation of proteins in HL-60, a human promyelocytic leukemia cell, in the presence of hydrogen peroxide [70]. After lipopolysaccharide [LPS] injury, mangiferin was described in vivo to induce brain protection. The mechanism of action included the induction of HO-1 and the interaction with Nrf2 pathway leading to inhibition of NLRP3 [71,72,73]. In vitro endothelial cells, which were stressed by a high-glucose medium, were protected by mangiferin with a decrease of TXNIP NLRP3 interaction and AMP-activated protein kinase (AMPK) function [74]. The induction of liver toxicity in vivo or in vitro by an endotoxin and D-galactosamine was controlled by mangiferin. An upregulation of Nrf2 and HO-1 and a downregulation of the expression of NLRP3, ASC, caspase-1 and Il-1β were measured [75]. Gartanin, present in mangosteen, among other xanthone derivatives, was reported to protect neurons against glutamate toxicity with modification of Nrf-2 only. No activity on HO-1 or sirtuin pathways was noticed, indicating an original mechanism of action [76].

Ginsenosides or panaxosides are a class of compounds purified from ginseng, an old traditional medicine. Considering ginsenoside Rg3, it appeared that antioxidant effects of this compound are stereospecific as demonstrated in the mouse after cyclophosphamide administration [77]. The R form appeared to be more active than the S one. This may indicate a specific binding site of the product who remains to be determined. The direct influence on inducible iNOS leading to s-nitrosylation of NLRP3 was also described as a possible mechanism of action with a concomitant decrease of ROS production [78].

Organosulfur compounds are reported to be present in various plants such as garlic, broccoli, Brussels sprouts, cabbages and arugula. For garlic extracts, a controversy exists on the usefulness of this family of compounds [79]. A protection against ischemia/reperfusion in rat kidney was described without a clear link with the inflammasome pathway [80]. For sulforaphane, antioxidant properties have been described with an induction of cytoprotective proteins and the opportunity to get patents [81]. Considering erucin, the description of the initial pro-oxidant activity on cancer cells is reported as the initial step of the induction antioxidant cellular defense by stimulation of antioxidant response element at the nuclear level [82]. Recently, a specific action of sulforaphane on NLRP3 and NLRC4 but not AIM2 in murine macrophages in vitro indicated a very interesting specificity, which deserves further experiments [83].

Whole extracts contain a large series of compounds that may be additive, synergic or antagonist. For example, the traditional use of *Nelumbo nucifera*, the sacred lotus, has allowed for the development of a series of analytical studies aiming to identify the active individual components. The initial extract is especially complex [84]. The presence of antioxidants such as flavonoids was observed, although many other structures have been identified, purified and tested. Nuciferine, an aporphine alkaloid, showed a strong inhibition of NLRP3 activation in a model of kidney inflammation [85]. However, nuciferine itself had a low antioxidant power [86].

Finally, experiments aiming to demonstrate synergy between active ingredients are also available in the literature. The major objective of this kind of work is to identify an originality for publication but also for patent filing. One example of this approach is the combined effect of nobiletin (a flavonoid from citrus species) and sulforaphane. A major activity on iNOS and HO-1 with concomitant decrease of IL-1β production was measured in murine macrophage cell line RAW264 [87]. Therefore, the selection of an appropriate mix may help to identify new therapeutic options, especially if the synergy of activity is really demonstrated. 

## 4. Conclusions

**Figure 2:**
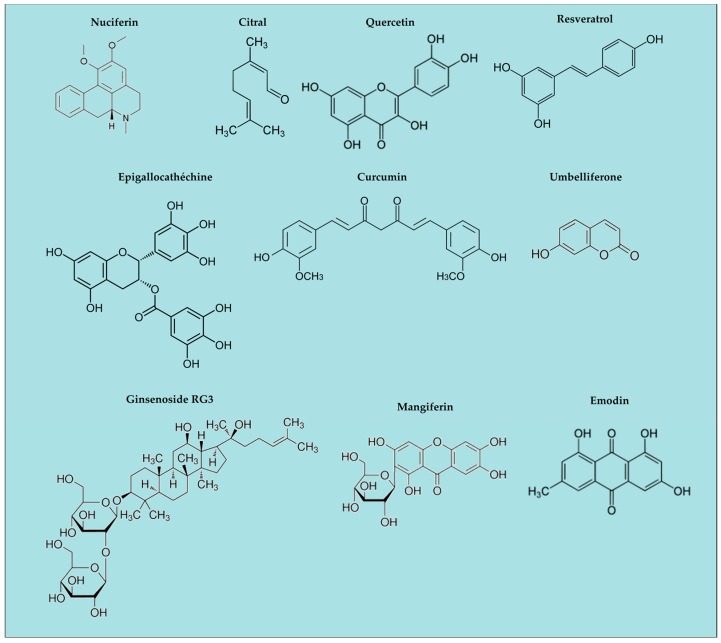
Natural ingredients with anti-inflammatory activity; examples of chemical structures.

**Figure 3:**
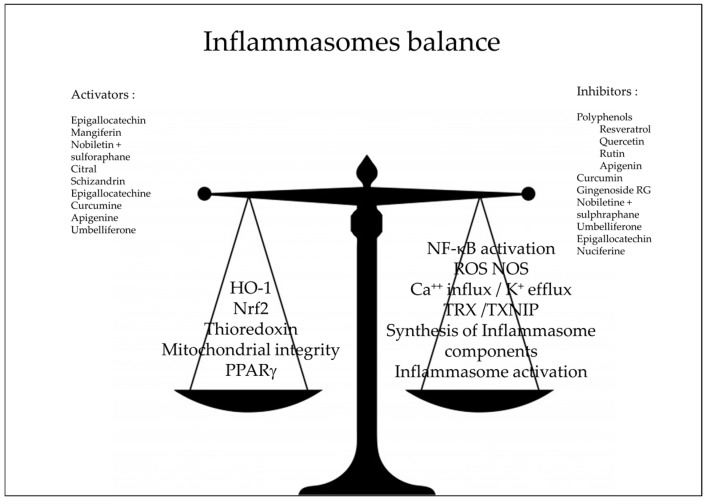
Natural antioxidants as inhibitors of inflammasomes pathway; NF-κB: Nuclear Factor-kappa B, HO-1: heme-oxygenase Nrf2 nuclear factor erythroid 2–related factor 2, ROS: reactive oxygen species, NOS: Nitric Oxyde Species, TXNIP: Thioredoxin Interacting Protein, TXR: Thioredoxin, PPARγ: peroxisome proliferator-activated receptors γ.

The overall landscape shows a great diversity of compounds (Figure 2) and corresponding targets (Figure 3). This may indicate that the anti-inflammatory activity is rather non-specific and that the inflammasomes are effectively the junction of many metabolic pathways.

The pharmacology of inflammasomes is a very interesting target for the pharmaceutical industry [88] and an original approach for the development of new compounds and the rejuvenation of marketed drugs or extracts used in traditional medicine. Numerous compounds are now described for their powerful activity as inhibitors and/or regulators of the pathway of the inflammasomes with many therapeutic possibilities. However, in the majority of cases, the demonstration of a direct link between the considered drug and the inflammasomes themselves is not available. When the anti-oxidant activity is studied in vitro in cellular or acellular conditions, a classification of compounds or mixtures according to their activity can be done. However, as soon as these compounds are administered orally or parenterally to a patient, there is no simple correlation between the initial antioxidant profiling and the final biological activity. The example of Oxygen Radical Absorbance Capacity (ORAC) illustrates perfectly the situation. ORAC value is well-known and used as a kind of calibrator for the anti-oxidant activities of compounds. Companies and final users are aware of it. However, the database on antioxidant profiling of foods with ORAC of the U.S. Department of Agriculture [89] is no longer available online due to the absence of a clear correlation between in vitro and in vivo activities. We can anticipate that the identification of a direct link between the antioxidant capacity and the final biological / therapeutic activity remains an elusive goal for a while. 

Two approaches may be considered. The first one may use an in silico approach to identify natural structures able to interact physically with inflammasomes before, during, and after signal 2 in order to identify specific linkers. This kind of approach was done for ferulic acid and will be developed as soon as all proteins of the inflammasome families will be described in detail. The second one may use a kind of “natural approach” by creating mixtures of natural antioxidants coming from different species, origins, parts etc., with complementary mechanisms of action as described for nobiletine and sulforaphane aiming to help the cell to solve the aggression and not to replace the cell machinery itself. In that case, the use of a more global approach such as an hemolytic test [90], in association with the multiplex analysis of a selected panel of cytokines to measure the influence of a drug on general antioxidant defense, may be more appropriate. 

## Figures and Tables

**Table 1:** Main families of Pattern Recognition Receptors.

Name	Localization	Ligands	Actions
TLR (1 to 13 ^1^)	Trans membrane (cell, endosome)	Pathogen fragments	NF-κB activation Production of immunomodulatory cytokines and chemokines
CLRs	Trans membrane or soluble	Carbohydrates	
NLRs (NODs)	Intracytoplasmic	Peptidoglycans	Production of IL-1β IL-18 Pyroptosis
RLHs	Intracytoplasmic	Ribonucleic acids	
AIMs	Intracytoplasmic	Deoxyribonucleic acids	

TLRs: Toll-like Receptors, CLRs: C-type Lectin Receptors, NLRs: Nucleotide-binding domain and Leucin-rich repeat Receptors, RLH: Retinoic acid-inducible gene Like Helicase Receptors, AIMs: Absent in Melanoma receptors. ^1^: number vary according to animal species.

**Table 2:** Patents for natural antioxidants acting on inflammasomes.

Composition	Biological Demonstration	Pathology Target	Reference
β hydroxybutyrate	Bone marrow derived macrophages	NLRp3 related pathologies	[25,26]
Ramalin	In vivo behaviour	Neurodegenerative diseases	[27,28]
*Sophora falvescens* extracts	THP-1 inflammation	Acute inflammation	[29,30]
Garlic extracts	Cell culture	Inflammation influenza	[31]
Fucoxanthinone	Cell culture	Inflammation immune disease	[32,33]
Mixture of drugs	Cell culture	Scleroderma	[34]
Glycyrrhizin and derivatives	Cell culture	Inflammation auto-immune diseases	[35,36]
